# Internalizing and Externalizing Problems, Empathy Quotient, and Systemizing Quotient in 4 to 11 Years-Old Siblings of Children with Autistic Spectrum Disorder Compared to Control Group 

**Published:** 2018-07

**Authors:** Farnaz Nasr Esfahani, Mitra Hakim Shooshtari, Rasoul Shirmohammadi Sosfadi, Fahimeh Saeed, Fereshteh Jalai, Aida Farsham, Reza Bidaki

**Affiliations:** 1 Mental Health Research Center, Tehran Institute of Psychiatry, School of Medicine, Iran University of Medicine Sciences, Tehran, Iran.; 2 Iran University of Medicine Sciences, Tehran, Iran.; 3 Institute of Cognitive Sciences Studies, Tehran, Iran.; 4 Clinical Psychology, Day Center in Roozbeh Hospital, Tehran University of Medicine Sciences, Tehran, Iran.; 5 Department of Psychiatry, Research Center of Addiction and Behavioral Sciences, Shahid Sadoughi University of Medicine Sciences, Yazd, Iran.; 6 Diabetes Research Center, Shahid Sadoughi University of Medicine Sciences, Yazd, Iran.

**Keywords:** *Autistic Spectrum Disorder*, *Child Behavior Checklist*, *Empathy Quotient*, *Systemizing Quotient*

## Abstract

**Objective:** This study was conducted to recognize the problems of living with a sibling with Autistic Spectrum Disorder (ASD) to improve their quality of life.

**Method** **:** A total of 30 participants were selected among the 4-to11- year-old siblings of children who had referred to Tehran Psychiatric Institute due to autism spectrum disorder. For the control group, 30 children aged 4 to11years old who were the siblings of patients with chronic diseases referring to Pediatric Clinic of Rasoul-e Akram (PBUH) hospital were selected. Gilliam Autism Rating Scale-Second Edition (GARS-2) was filled out for patients and siblings participating in the study and Child Behavior Checklist was completed by their parents.

**Results: **The mean age of the patients in this study was 4.46 ± 9.66 years (range: 1.5-22 years), and the mean age in the healthy children was 2.54 ± 8.18 years (range: 4-11 years). The mean scores of anxiety/depression, withdrawn/depressed, somatic complaints, social problems, thought problems, attention problems, and rule-breaking behavior subscale of CBCL (Child Behavior Checklist) were not significantly different between groups. Aggressive behavior was the only subscale that showed such difference (p = 0.008). Externalizing problems in children who had siblings with ASD was higher than children who had siblings with physical illness. In a group in which a sibling had ASD, sisters were more anxious/ depressed than brothers.

**Conclusion: **Due to various psychological and social problems that siblings of children with ASD experience throughout their life, studying their psychological problems to improve their quality of life seems to be of paramount importance.

Autism is a developmental disorder, which is a part of a more general class of pervasive developmental disorders or autism spectrum disorders (ASD). Autism prevalence in different countries has been reported differently. 

 Fombone’s best estimate of the prevalence rate of ASD is reported to be between 60 and 70 per 10 000 according to recent measurements (1). 

Approximately, 1 in every 800 to 1000 children has autism based on autism definitions, but some autism symptoms can be seen in 1 out of 150 individuals (kaplan & sadock’s comprehensive textbook of psychiatry 2017).

In Iran, Samadi screened more than 1.3 million four-year- old children for autism over three academic years. He used Social Communication Questionnaire (SCQ) as a screening tool and Autism Diagnostic Interview-Revised (ADI-R) to confirm the diagnosis. The resulting prevalence was 6.26 per 10 000 for typical autism (2). 

Children with autism show shortcomings in a particular aspect of social cognition, particularly empathy. The ability of people to react in sympathetic ways depends on both social ability and understanding the mental state of the person (thoughts, beliefs, and feelings) that have provoked their behavior and the adoption of an appropriate emotional response.

The ability to empathize enables people to correctly interpret social situations and respond in a proper manner. Theory of mind appears in three- to five- year old children with normal growth. However, children with autism show specific injury. In fact, children with autism show mind-blindness, so they often cannot understand that others have psychological states and these states are causing and directing their actions (3). 

ASD can lead to social isolation, difficulty in receiving treatment, medical problems, difficulties in maintaining jobs, and financial burden for the family (4). Different studies conducted on parents of children with ASD in Iran found that these parents, compared to the parents of normal children, suffer higher levels of stress and mental health problems (5). The level of stress that parents of children with ASD experience is influenced by the resources at their disposal; parents who receive appropriate social support can more successfully cope with the problems associated with raising a child with ASD (6). 

Various studies have examined the psychological and social factors in siblings of children with autism. According to Dillon, if a child lives with a sibling who has autism spectrum in a family, the child with an autism spectrum disorder will receive a disproportionate share of parental attention (7). In a study on children in families that have children with intellectual disabilities or autism spectrum disorder, Schuntermann concluded that siblings of patients with ASD fight for their independence to create balance between their needs and those of their siblings (8).

Gold examined depression and social adjustment in siblings of boys with ASD (1993). The results of the study indicated that children who have a brother with ASD had a higher score of depression compared to the control group. However, this difference was not perceived in the social adjustment (9).

Ward et al. studied the experiences of siblings with ASD using a qualitative-descriptive study on 11 brothers and 11 sisters with ASD (10). The results revealed that the participants recognized the negative aspects of life (reduced parental attention, responsibility, annoying behavior, and communication problems) and the positive aspects of life (empathy, love and encouraging children, and understanding changes in life) in a person with ASD. The brothers often wished that they could play more with their sibling. They repeatedly mentioned aggressive behavior in children or adolescents. While the sisters emphasized the aspects of relationship and communication problems with their sibling with the disorder.

In their study, Verte et al. concluded that the potential of compatibility issues in the population of patients with ASD is not higher than their siblings without the disorder (11). The results of the study suggest that children with brothers and sisters, especially those aged 6 to 11 years, had more behavioral problems compared to the control group. Sisters of children with the disorder attributed higher social competence to themselves. Sisters of children with the disorder aged 12 to 16 years had more positive self-concept. In both groups, siblings with more negative self-concept were less likely to have social skills. Conversely, siblings with more positive self-concept gained better results in the social field.

Ross et al. (2006) conducted a study to assess the compatibility problems and coping strategies in siblings of children with autism spectrum disorder (12). Aggressive behaviors were the most common oppositional problems and anger was the most common response. Siblings of children with ASD generally did not blame themselves or others to deal with problems of their siblings with the disorder. The coping strategy and the knowledge about autism spectrum disorder did not correlate with the compatibility. Based on Child Behavior Checklist, 40% of the siblings of children without the disorder had marginal or clinical problems. One study found that the siblings with autism spectrum are at increased risk of internalizing behavior problems.

 Due to various psychological and social problems that siblings of children with ASD experience throughout life, studying their psychological problems to improve their quality of life is of significant importance.

## Materials and Methods

This was an analytical cross-sectional study. The study population was selected among the 4- to11- year-old siblings of children who had referred to Tehran Psychiatric Institute and had been visited by a child psychiatrist after a diagnosis of autism spectrum disorder. A total of 30 children participated in this study. We selected every sibling of autistic children who aged 4 to11 years.

 For the control group, 30 children were selected from 4- to11-year-old siblings of children with chronic diseases referring to Pediatric Clinic of Rasoul-e Akram (PBUH) hospital. 

A disease or condition is considered to be chronic in childhood under the following conditions: (1) If it occurs in children aged 0 to 18 years; (2) the diagnosis is based on medical scientific knowledge and can be established using reproducible and valid methods or instruments according to professional standards; (3) if it is not yet curable, or for mental health conditions, if it is highly resistant to treatment; and (4) if it has been present for longer than three months or if it lasts longer than three months, or it has occurred three times or more during the past year and will probably reoccur. This definition was operationalized using the ICD-10 classification of the World Health Organization (WHO; International Statistical Classification of Diseases and Related Health Problems (ICD) , 10th revision, Geneva, Switzerland, 1992) (Defining chronic diseases and health conditions in childhood (0–18 years): national consensus in the Netherlands Lidwine B. Mokkink & Johanna H. van der Lee & Martha A. Grootenhuis & Martin Offringa & Hugo S. A. Heymans & The Dutch National Consensus Committee “Chronic Diseases and Health Conditions in Childhood”).

First, Gilliam Autism Rating Scale-Second Edition (GARS-2) was filled out for patients and siblings participating in the study.

Then, Child Behavior Checklist was completed by the parents about the siblings of the child with ASD. This questionnaire is used to analyze different behavioral aspects including adaptive behavior and behavioral problems at different ages. Also, empathizing-systemizing test was completed about the siblings of the child with ASD. Child Behavior Checklist and empathizing-systemizing test were completed for these children.

This scale measures the severity of ASD (13). Samadi evaluated the use of GARS in Iran. The Factor analysis broadly confirmed the three subscales, all of which had high internal consistency. Individuals with autism were clearly distinguished from the other two groups and a cut-off score was identified that maximized the scale's sensitivity and specificity (14).

Empathizing-systemizing test has 55 items and parents can choose from four options. The parents indicate how strongly they agree with each statement about their child. A total of 27 items are allocated to empathy quotient with the maximum score of 54. Furthermore, 28 items are allocated to empathy quotient with the maximum score of 58. Questionnaires with five or more blank items were considered incomplete, and these data were discarded in subsequent analyses. This questionnaire was translated into Persian by Maryam Jalali et al. in 2011; the reliability of the 55 items is 0.7 using Cronbach's alpha coefficient (15).

Child Behavior Checklist refers to one component of the Achenbach System of Empirically Based Assessment (ASEBA) and measures aggressive behavior, anxiety/depression, attention problems, rule-breaking behavior, somatic complaints, social problems, thought problems, and withdrawn/depressed. This questionnaire measures emotional-behavioral problems as well as academic and social abilities and competencies of 6 to 18 year-old children from parents’ perspectives, and it typically takes 20 to 25 minutes to complete. This questionnaire includes 115 questions, addressing a variety of children's behavioral states. Responses are recorded on a Likert scale with three options ranging from 0 to 2. Accordingly, the score of 0 is attributed to those items which are not perceived in child's behavior, score of 1 is allocated to the attitudes and behaviors that are sometimes perceived in the child, and the score of 2 is given to items that are almost always or always perceived in child’s behavior. This questionnaire was normalized by Tehrani-Doost in Iran (16). In addition, a form containing demographic data was completed for each participant in the study. Parents in the study were given the necessary explanations and their consent was obtained.

The results for quantitative variables were defined as mean and standard deviation (mean ± SD), and for qualitative variables, they were expressed as percentage. Comparisons between quantitative variables were stated by t test and Mann–Whitney U test was performed in case of abnormal distribution. The comparison between qualitative variables was performed using Chi-square or Fisher's exact test. Statistical analysis was done using SPSS Version 21. Significance level was considered to be less than 0.05.

## Results

This study included 30 children, whose siblings had been suffering from physical illness, and 30 children whose siblings had ASD. Information on demographic variables is demonstrated in Table 1. Of the participants of the ASD group, 53.3% were male and 46.7% female; this percentage in physical illnesses was 33.3% in males and 66.7% in females (p = 0.118). Of the individuals with ASD, 80% were male and 20% female. Also, in the physical illnesses group, 46.7% were male and 53.3% female.

In this study, 96.7% of the parents were living together and 98.3% of healthy children lived with the child with the illness. The mean age of the patients in this study was 4.46 ± 9.66 years (range: 1.5-22 years), and the mean age in the healthy children was 2.54 ± 8.18 years (range: 4-11 years).

According to the results of GARS, children who had previously been diagnosed with ASD by a psychiatrist were placed within the clinical ASD. However, based on the results of the scale, none of their siblings were diagnosed with ASD. 

The mean score of anxiety/depression was 5.16 ± 4.63 in the case group and 4.5 ± 4.47 (p = 0.48) in the control group; the mean of withdrawn/depressed was 2.61 ± 2.9 in the case group and 2.03 ± 2.07 (p = 0.159) in the control group; somatic complaints was 1.5 ± 1.9 in the case group and 1.86 ± 4.61 (p = 0.124) in the control group; social problems was 3.36 ± 3.13 in the case group and 3.23 ± 2.69 (p = 0.994) in the control group; thought problems was 1.7 ± 2.47 in the case group and 1.46 ± 1.92 (p = 0.936) in the control group; attention problems was 2.7 ± 3.13 in the case group and 2.4 ± 2.58 (p = 0.97) in the control group; rule-breaking behavior was 2 ± 2.36 in the case group and 1.5 ± 1.83 (p = 0.38) in the control group; aggressive behavior was 9.31 ± 6.27 in the case group and 5.51 ± 5.23 (p = 0.008) in the control group (Table 1). In fact, aggressive behavior in children who had a sibling with ASD was more than in children who had a sibling with a chronic physical illness.

The mean score of internalizing was 9.56 ± 7.25 in the case group and 8.4 ± 9.53 (p = 0.23) in the control group, and the mean score of externalizing was 11.1 ± 8.05 in the case group and 7.06 ± 6.8 (p = 0.027) in the control group. Externalizing problems in children who had siblings with ASD was higher than in children who had siblings with physical illness. The mean score of systemizing quotient test was 19.36 ± 5.93 in the case group and 19.6 ± 7.52 (p = 0.689) in the control group, and the mean score of the empathy quotient test was 37.66 ± 6.55 in the case group and 40.1 ± 7.89 (p = 0.257) in the control group (table2).

The mean scores of various subscales of child behavior checklist in the case and control groups are shown in Table 3 based on the gender of the healthy child. There was a statistically significant relationship between the mean score of anxiety/depression subtype and gender in healthy children from the case group. In fact, in a group in which a sibling had ASD, sisters were more anxious/depressed than brothers. The mean score of empathy quotient was 40.9 in sisters of patients with ASD and it was 38.5 (p = 0.076) in brothers of patients with ASD. The mean score of systemizing quotient was 19.45 in sisters of patients with ASD and it was 19.9 (p = 0.404) in brothers of patients with ASD. The mean score of empathy quotient was 39.64 in sisters of patients with physical illness and it was 35.94 (p = 0.502) in brothers of patients with physical illness. The mean score of systemizing quotient was 19.79 in sisters of patients with physical illness and it was 19 (p = 0.99) in brothers of patients with physical illness. 

As presented in Table 4, the mean difference of anxiety/depression in a healthy sister and healthy brother was statistically significant in brothers with ASD (p = 0.012).

In fact, the level of anxiety and depression was higher in a healthy sister of a brother with ASD than in a healthy brother of a brother with the disorder. On the other hand, the mean difference of internalizing score was statistically significant in a healthy sister and healthy brother of the boys with autism spectrum disorder (p = 0.011). The mean score of internalizing score in a healthy sister of a brother with ASD was higher than in a healthy brother of a brother with the disorder.

As demonstrated in Table 5, no significant differences were observed in the average of different subscales of Child Behavior Checklist in the group of participants with physical illness (control), based on the gender of the healthy siblings.

**Table 1:** Frequency and Percentage of Demographic Variables in Both Groups

**Demographic Variables**			**Medical Disease Group (Control)**			**Autistic Group (Case)**		**P-Value**
			**Frequency**		**Percentage**	**Frequency**	**Percentage**	
Gender of the patient	boy		14		46/7	24	80	0.007
	girl		16		53/3	6	20	
Gender of sibling	boy		10		33/3	16	53/3	0.118
	girl		20		66/7	14	46/7	
Medical treatment	yes		30		100	18	60	<0.0001
	no		0		0	12	40	
Non pharmaceutical treatment	yes		12		40	24	80	0.002
	no		18		60	6	20	
Father’s education	illiterate		1		3/3	0	0	0.369
	primary		2		6/7	4	13/3	
	diploma		22		73/3	16	53/3	
	bachelor		2		10	6	20	
	more		2		6/7	4	13/3	
mother’s education	illiterate		2		6/7	0	0	0.263
	primary		5		16/7	4	13/3	
	diploma		19		63/3	18	60	
	bachelor		2		6/7	7	23/3	
	more		2		6/7	1	3/3	
Living both parents together		yes		29	96/7	29	96/7	
		no		1	3/3	1	3/3	
Living affected child with healthy sibling		yes no		29 1	96/7 3.3	30 0	100 0	0.313

**Table 2:** Mean and Standard Deviation of All Subscales of CBCL, Empathy Quotient and Systemizing Quotient in Both Groups

**Subscales**	**Autistic Group (Case)**		**Medical Disease Group ** **(Control)**		**P-Value**
	**Mean**	**SD**	**Mean**	**SD**	
Anxiety/depression	5.16	4.63	4.5	4.47	0/48
Withdrawal/depression	2.9	2.61	2.03	2.07	0/159
Somatic complaints	1.5	1.9	1.86	4.61	0/124
Social problems	3.36	3.13	3.23	2.69	0/994
Thought problems	1.7	2.47	1.46	1.92	0/936
Attention problems	2.7	3.13	2.4	2.58	0/970
Rule-breaking behavior	2	2.36	1.5	1.83	0/380
aggressive behavior	9.31	6.27	5.51	5.23	0/008
Internalizing	9.56	7.25	8.4	9.53	0/230
Externalizing	11.1	8.05	7.06	6.8	0/027
systemizing quotient	19.36	5.93	19.6	7.52	0/689
empathy quotient	37.66	6.55	40.1	7.89	0/257

**Table3:** Mean and Standard Deviation of All Subscales of CBCL, Empathy Quotient and Systemizing Quotient in Both Groups Based on Healthy Child’s Gender

**Subscales**	**Autistic Group (Case)**		**P-Value**	**Physical Illness Group (Control)**		**P-Value**
	**Girl**	**Boy**		**Girl**	**Boy**	
Anxiety/depression	7	3.56	0.041	4.75	4	0.746
Withdrawal/depression	3.64	2.25	0.27	2	2.1	0.779
Somatic complaints	1.5	1.5	0.862	1.95	1.7	0.650
Social problems	4	2.81	0.447	3	3.7	0.397
Thought problems	2.57	0.9	0.25	1.35	1.7	0.502
Attention problems	3.21	2.25	0.593	2.45	2.3	0.880
Rule-breaking behavior	2.38	1.69	0.734	1.6	1.3	0.812
aggressive behavior	10.21	8.47	0.456	5.4	5.78	0.417
Internalizing	12.14	7.31	0.128	8.7	7.8	0.984
Externalizing	12.23	10.13	0.627	7	7.22	0.650
systemizing quotient	40.9	38.5	0.76	39.64	35.94	0.502
empathy quotient	19.45	19.9	0.404	19.79	19	0.99

**Table 4:** Mean of All Subscales of CBCL in ASD Group Based on Healthy Child’s Gender and Gender of Autistic Child

**Subscales**		**Autistic group (case)**				**P-Value**
	**Boy with ASD**		**P-Value**	**Girl with ASD**		
	**Healthy Brother**	**Healthy Sister**		**Healthy Brother**	**Healthy Sister**	
Anxiety/depression	3/33	8/08	0/240	4/25	0/5	0/012
Withdrawal/depression	2/16	4/25		2/5		0/103
Somatic complaints	1/16	1/58	0/634	2/5	1	0/545
Social problems	2/25	4/58	0/1	4/5	0/5	0/1
Thought problems	0/9	2/83	1	1	1	0/245
Attention problems	2/16	3/75		2/5		0/261
Rule-breaking behavior	1/5	2/45	0/812	2/25	2	0/567
aggressive behavior	7/16	11/16	0/564	13/6	4/5	0/172
internalizing	6/66	13/91	0/1	9/25	1/5	0/011
externalizing	8/66	13/27	0/374	16	6/5	0/308

**Table 5:** Mean of All Subscales of CBCL in Group with Physical Illness Based on Healthy Child’s Gender and Gender of Child with Physical Illness

**Subscales**	**Group of Physical Illness**					**P-Value**
	**Boy with Physical Illness**		**P-Value**	**Girl with Physical Illness**		
	**Healthy Brother**	**Healthy Sister**		**Healthy Brother**	**Healthy Sister**	
Anxiety/depression	5	5/3			4/5	0/558
Withdrawal/depression	2/5	2/33	0/367	0/5	1/85	0/511
Somatic complaints	2	5/16	0/929	0/5	0/57	0/628
Social problems	4/25	3/16	0/52	1/5	2/92	0/362
Thought problems	2	2/33	0/93	0/5	0/92	0/842
Attention problems	2/87	1/83			2/71	0/507
Rule-breaking behavior	1/5	2	0/557	0/5	1/42	0/841
aggressive behavior	6/37	7/33	0/347	1	4/5	0/793
internalizing	9/5	12/83	0/103	1	6/92	0/897
externalizing	7/87	9/33	0/415	2	6	0/845

## Discussion

In the present study, out of the examined subscales in the Child Behavior Checklist, only the difference between subscale of aggressive behavior and externalizing score was statistically significant higher in siblings of children with ASD and the control group. This level in the sample group was higher than in the control group.

In this study, quality of life was not investigated, but most subscales of Child Behavior Checklist in the group who had a sibling with ASD were similar to siblings of children with chronic physical illness. However, in the subscale of aggressive behaviors, this level was higher in patients with ASD than physical illness. Given that aggressive behaviors are common in children with autism and that the sibling of the patients in this study and almost 99% of healthy children were living with the child with disorder, they could imitate aggressive behaviors from the child with ASD. This can be due to the fact that the siblings of children with ASD are themselves exposed to aggression. According to previous studies, autistic disorder is associated with parents’ exhaustion and stress (7, 17). Parents’ high level of pressure causes more stress, depression, and anxiety in them. Autism has also a negative impact on family functioning and relationships between spouses, the effect of which is more than that of the parents of children with mental and physical disabilities and development problems (18). Glidden and Jobe (2007) found that parents of such children have endured many concerns and difficulties (19). Parents of patients with ASD have more economic concerns, higher rate of job loss, and higher medical costs compared to parents of children with other disabilities (25). Different aspects of quality of life, such as social activities, family exhaustion, school, independence, and parents' concerns about the quality of life of children with ASD, have been studied. Based on socioeconomic status, previous participation in the group supporting siblings, parental stress, family habits, problem-solving power, and establishing family relationships predict siblings’ adjustment disorders (20). According to Lee, quality of life in children with ASD and their families is lower than in families of children with ADHD, and the control group of unaffected children (21). In a study by Rao on 15 parents of children with ASD with high functioning and a control group, it was found that parents of children with ASD with high functioning experience expressively higher levels of stress than the parents who do not have a child with psychiatric disorders (18).

With regards to the situation of families of children with ASD in Iran, one can predict that the quality of life in these families is low; this is especially true in the families studied in this research, as these families are often from a low socioeconomic status. In Iran, families of children with ASD lack support groups and are often forced to solve problems alone. Clinical experience shows that many of these families are trying to take their children to social and family gatherings less than usual. Ignorance and misconceptions regarding the severity of this disorder increase with such isolation. Most of these parents suffer the feelings of inferiority and incompetence in gatherings and attribute the problems of their children to their own failure.

Ross et al. (2006) studied compatibility, problems, and coping strategies in siblings of children with autism spectrum disorder, in which aggressive behaviors were the most common oppositional problem and anger was the most common response (12).

Paying too much attention to these children provokes anger in their siblings. They feel a sense of resentment and hatred towards their ill brother or sister. Such feelings along with the feeling of guilt because of their health may lead to complex emotional experiences in siblings of ill children. As the average age of these children was 2.74 ± 7.93 years, they have concrete form of thinking and are not able to easily express their feelings verbally. Lack of their familiarity with their feelings on the one hand and not being allowed to express feelings on the other could result in aggressive behavior and externalizing scores.

Iranian mothers may play the role of the victim in dealing with such a complicated illness and they try to devote their whole life to the child with the disorder (22). It seems that in such circumstances they have less time to communicate with the healthy child. As mothers in ABA play the role of a therapist assistant at home and have to fulfil their stereotypical role as a housewife, they experience a higher level of stress and fail to pay attention to their own and other family members’ needs.

On the other hand, instilling a sense of guilt to the mother for the developmental delay in the child by other family members can impose more emotional distress on them, making it harder to communicate with other children.

In some families, healthy children with a role reversal would have to do some housework and, in some cases, receive instructions or care for the child with the disorder.

Based on the results, anxiety-depression in sisters of a sibling with ASD was higher than in brothers of a sibling with ASD. The high level of symptoms in the girls can be caused by psychological and cultural differences between the 2 groups. Girls paiy more attention to details and to the exciting relationships prevailing among people, moreover, different role expectations of the society from a girl could account for their higher vulnerability level. Moreover, girls spend more time at home, are more affected by family issues, and are biologically more prone to anxiety and depression.

In a study conducted by R. Giallo and S. Gavidia-Payne in 2006 on 49 children and adolescents who had siblings with disability in different areas, it was confirmed that factors about the personal experience of siblings from stress and adaptation are stronger predictors of adjustment disorders in siblings (23). In the present study, parents were studied for psychiatric problems. The existence of the respective factor can also affect the internalization and externalization problems in healthy children. Different studies conducted on parents of children with ASD in Iran indicated that these parents have higher levels of stress and problems related to mental health compared to the parents of healthy children (4). The level of stress that parents of children with ASD experience is affected by the resources at their disposal (5, 6). Parents who receive appropriate social support can more successfully cope with the problems associated with raising a child with ASD (24).

A qualitative study was conducted by Benderix (2000) on the experience of having a sibling with ASD or mental retardation, which was performed among 14 siblings in 5 families with ASD children or mental retardation. Using qualitative content analysis, 7 content categories were obtained: (1) premature responsibility, (2) lamenting, (3) exposure to frightening behaviors, (4) empathy, (5) hope for the liberation of a group home, (6) feelings of insecurity and anxiety as a result of physical violence, and (7) the negative effect on relationships with friends (25) . No relationship was found in other subscales and empathizing-systemizing test. The need for this study was that the siblings of these children could be examined in terms of their different needs compared to other children who had a sibling with severe physical illness.

## Limitation

The sample size of this study was small. Moreover, in this study, siblings were evaluated only for having ASD, and we did not have any information about other psychiatric disorders that have aggression as their presentation.

## Conclusion

According to the obtained results, aggressive behavior and externalizing scores in siblings of children with ASD were more than siblings of children with physical illness. On the other hand, anxiety and depression were higher in a healthy sister with an ASD brother than in a healthy brother of a brother with the disorder. Also, internalizing score was higher in a healthy sister of an ASD brother than in a healthy brother of a brother with the disorder. Therefore, similar to the presence of chronic physical diseases in other children of the family, the presence of a child with ASD in the family has an impact on the development of the psychological symptoms, such as anxiety/depression, aggressive behavior, and externalizing scores in healthy children. Considering the importance of this issue, we highly suggest conducting qualitative studies on this topic in the future.
